# Pandora's box

**DOI:** 10.1192/bji.2024.19

**Published:** 2024-08

## ‘Is there anybody there?’

The Covid pandemic highlighted how detrimental social isolation can be to us humans. In November 2023, recognising loneliness as a ‘pressing threat to health’, the World Health Organization (WHO) launched a new commission to foster social connection as a health priority and encourage and expedite the development of solutions in all countries (high, middle or low income) across the world. According to the WHO, one in four older people and 5–15% of adolescents experience isolation and loneliness, and these figures are an underestimate.

Living alone is increasingly common in the European Union (data between 2009 and 2020), and in the USA people have decreased the time they spend with family, friends neighbours and co-workers (survey between 2003 and 2020). Vivek Murthy, US Surgeon General and member of the WHO Commission on loneliness, declared an ‘epidemic’ of loneliness. A school survey of 13–17-year-olds, in 70 countries, between 2002 and 2018, found that 11.7% felt lonely most of the time or always in the past year. The evidence for chronic isolation and loneliness has worryingly been found to be associated with increased risks of heart disease, diabetes, dementia and depression, as well as being an independent predictor of premature death. There are various reasons for these outcomes, including not only psychophysiological mechanisms but also access to healthcare and information, lifestyle and the role of social media.

How do we define loneliness and does it equal social isolation? Being alone doesn't always mean one is feeling lonely. One can feel lonely in the company of others. Is social connection a replacement, a solution for loneliness and isolation? Probably yes, in some cases, but it is not a panacea.

Servick K. Striving to connect. Science 2024. Available from: https://doi.org/10.1126/science.zf8vutm.

Welch V, Tanjong Ghogomu E, Dowling S, Choo WY, Yunus RM, Mohd TAMT, et al PROTOCOL: in-person interventions to reduce social isolation and loneliness: an evidence and gap map. Campbell Syst Rev 2023; 19(3): e1340.37361556 10.1002/cl2.1340PMC10286723

## Do not forget the children

Pandora has in the past reported on US studies on drug overdosing and the higher rates among some ethnic groups. What was not highlighted in such publications was the impact of the loss of a parent on children and the need to take this into consideration in any management programmes. Despite the profound and long-term effects on children of the loss of a parent to a drug overdose, little is known in the US about the numbers concerned. A recently published collaborative study between the National Institutes of Health, National Institute on Drug Abuse, the Substance Abuse and Mental Health Services Administration and the Centers for Disease Control and Prevention set out to look at this. The authors specifically aimed to estimate the number and rate of children losing a parent to a drug overdose from 2011 to 2021.

Data from the National Survey on Drug Use and Health and the National Vital Statistics System were used in a cross-sectional study stratified by parental age, gender, race and ethnicity. Around 650 000 adults aged 18 to 64 years with a mean age of 41 years died between 2011 and 2021, with twice as many men as women; 75% of the total were non-Hispanic Black people. A total of 321 566 community-dwelling children lost a parent. Twenty-seven per 100,000 children lost a parent in 2011, with the number climbing to 63 per 100,000 in 2021. The highest rates of increase were found among non-Hispanic American Indians or Alaskan Natives (187%).

The authors suggest that any policy and programme planning take into consideration the burden of drug overdoses on the family and children. They point out the need to specifically address the socioeconomic, educational and healthcare needs of the affected children.

Jones CM, Zhang K, Han B, Guy GP, Losby J, Einstein EB, et al Estimated number of children who lost a parent to drug overdose in the US from 2011 to 2021. JAMA 2024; 81(8): 789–96.10.1001/jamapsychiatry.2024.0810PMC1107978738717781

## Neurodevelopmental disorders and left-handedness

It is well known that handedness is determined by our brain hemisphere asymmetry, with left dominance that presents as 90% of us being right-handed and the other 10% being left-handed. In case you wonder, there is no difference between countries or ethnicities, and there has been no significant change in these figures over the years. Differences observed of 2–14% in different parts of the world are thought to be due to enforcing right hand use in some cultures. Handedness is determined at a very early stage of development and is already present at 10 weeks of gestational age. It has been known for some time that there is about 30% heritability of structural and functional brain symmetry, particularly in relation to language, lateralised to the left hemisphere.

Data from meta-analyses show that left-handedness is higher among individuals with neurodevelopmental disorders such as autism as well as those with schizophrenia and Parkinson's disease. The majority of left-handed people, however, do not have any such disorders. Attention has been drawn to rare coding variants, known to be involved in the genetic makeup of neurodevelopmental disorders and these may be responsible for the minority of left-handed people with such conditions. The authors of a recent study, using the UK Biobank general population data-set for exome-wide screening and burden heritability analysis, identified the role of rare coding variants in left-handed people. They claim this finding offers potential insights not only into the mechanism of left–right axis formation in the brain but also genetic susceptibility to brain disorders.

Schijven D, Soheili-Nezhad S, Fisher SE, Francks C. Exome-wide analysis implicates rare protein-altering variants in human handedness Nat Commun 2024; 15: 2632.38565598 10.1038/s41467-024-46277-wPMC10987538

## Restoring bilingual speech – artificial intelligence to the rescue

Several neurological conditions including stroke and motor neurone disease can cause anarthria, loss of the ability to articulate language. Various speech–brain–computer interfaces have been developed to restore language communication for those affected. Research on decoding speech from brain activity has so far focused on single languages, although two-thirds of the world population are currently proficient in two languages. What perhaps matters most to us all as individuals is to retain our mother tongue, a connection with our roots and a link to our identity.

A recent publication examined the production of bilingual speech in an individual, bilingual in Spanish and English, who became anarthric as a result of vocal tract and limb paralysis. Using electrocorticography with deep learning and statistical natural language models of both languages, they recorded and decoded activity from the person's speech–motor cortex. Their findings indicated shared cortical articulatory representations, which persisted despite the paralysis. This allowed the decoding of multiple languages without the need to train separate language specific decoders. Following this, they were able to develop an artificial-intelligence-powered device that could read brain speech; when implanted in the brain, this enabled the paralysed bilingual individual to speak in both his native and his second language.

Silva AB, Liu JR, Metxger SL, Bhaya-Grossman I, Dougherty ME, Seaton MP, et al A bilingual speech neuroprosthesis driven by cortical articulatory representations shared between languages. *Nat Biomed Eng* [Epub ahead of print] 20 May 2024. Available from: 10.1038/s41551-024-01207-5.PMC1155423538769157

## Eucalyptus keeps the mosquitos away

Are you one of those people that mosquitos make a beeline for, but you can't understand why? Well, we now know it is to do with certain chemicals that determine your body odours and – almost like pheromones – draw the mosquitos to you. In a large-scale study in Zambia using a multi-choice preference assay with infrared motion vision under semi-field conditions, researchers determined the Zambian Anopheles mosquito's preferences for human skin. These mosquitoes liked warm human skin at night, with ample concentrations of the volatile carboxylic acids butyric acid, isobutyric acid, isovaleric acid and methyl ketone acetoin, the latter generated by skin microbes. By contrast, they stayed away from body odour that lacked carboxylic acids, and the presence of monoterpenoid eucalyptol seemed to put them off.

Giraldo D, Rankin-Turner S, Corver A, Tauxe GM, Gao AL, Jackson DM, et al Human scent guides mosquito thermotaxis and host selection under naturalistic conditions. Curr Biol 2023; 33(12): 2367–82.37209680 10.1016/j.cub.2023.04.050PMC10824255

